# Microplastics in sea ice and seawater beneath ice floes from the Arctic Ocean

**DOI:** 10.1038/s41598-020-61948-6

**Published:** 2020-03-19

**Authors:** La Daana K. Kanhai, Katarina Gardfeldt, Thomas Krumpen, Richard C. Thompson, Ian O’Connor

**Affiliations:** 1 Marine and Freshwater Research Centre, Galway Mayo Institute of Technology, Galway, Ireland; 2 Marine Biology and Ecology Research Centre, University of Plymouth, Plymouth, United Kingdom; 3 Department of Chemistry and Chemical Engineering, Chalmers University of Technology, Göteborg, Sweden; 4 Alfred Wegener Institute, Helmholtz Centre for Polar and Marine Research, Bremerhaven, Germany

**Keywords:** Marine biology, Physical oceanography

## Abstract

Within the past decade, an alarm was raised about microplastics in the remote and seemingly pristine Arctic Ocean. To gain further insight about the issue, microplastic abundance, distribution and composition in sea ice cores (n = 25) and waters underlying ice floes (n = 22) were assessed in the Arctic Central Basin (ACB). Potential microplastics were visually isolated and subsequently analysed using Fourier Transform Infrared (FT-IR) Spectroscopy. Microplastic abundance in surface waters underlying ice floes (0–18 particles m^−3^) were orders of magnitude lower than microplastic concentrations in sea ice cores (2–17 particles L^−1^). No consistent pattern was apparent in the vertical distribution of microplastics within sea ice cores. Backward drift trajectories estimated that cores possibly originated from the Siberian shelves, western Arctic and central Arctic. Knowledge about microplastics in environmental compartments of the Arctic Ocean is important in assessing the potential threats posed by microplastics to polar organisms.

## Introduction

Within the past decade, microplastic pollution emerged as an issue of concern in the Arctic Ocean due to the discovery of these contaminants in its sea ice^1,2^, surface and sub-surface waters^3–8^, deep sea sediments^9–11^, biota^4,6,12,13^ and mostly recently its snow^14^. Of the environmental compartments in this remote oceanic basin, it was shown that sea ice can function as a temporal sink, a secondary source and a transport medium for microplastics^1,2^. Historically, however, observational records of ‘dirty ice’, examination of Arctic ice cores, laboratory-based experiments and modelling studies were the first to highlight the potential for sea ice in the Arctic Ocean to trap, transport and redistribute sediments and various contaminants (i.e. metals, organochlorines, organophosphates, polycyclic aromatic hydrocarbons)^15–27^. Microplastics (plastic particles <5 mm in diameter) were first discovered in sub-sections of 4 ice cores retrieved from various locations in the Arctic Ocean^1^. In this initial study, a total of 6 types of synthetic polymers were reported in the ice cores and microplastic concentrations in Arctic sea ice were estimated (based on extrapolations) to be between (1.3–9.6) × 10^4^ particles m^−3^. A second study^2^, subsequently examined 5 sea ice cores from the Arctic Ocean, reported on the presence of smaller (<100 µm in diameter), more diverse types of synthetic polymers (n = 17) and estimated (based on extrapolations) microplastic concentrations in Arctic sea ice to range between (1.1 × 10^6^)–(1.2 × 10^7^) particles m^−3^. Both studies^1,2^, while limited in scale and extent, collectively suggested that sea ice can function as a sink, source and transport medium for microplastics in the Arctic Ocean.

Within this remote oceanic basin, microplastic entrapment within sea ice potentially occurs during sea ice formation and drift while microplastic release occurs upon the melting of sea ice^1,2,14^. In the Arctic Ocean, while sea ice formation occurs over the central Arctic Ocean, the most important regions for this are the shallow Siberian shelves in the Eurasian Basin and the Beaufort Sea in the Amerasian Basin^15^. Microplastics entrapped within sea ice during its formation can potentially be reflective of microplastics present in seawater from known ice formation regions in the Arctic^1,2^. While the majority of sea ice melts in close proximity to its formation zone, some sea ice (i.e. that which forms over the Siberian shelves) is advected off the coast, joins the Transpolar Drift with some eventually exiting through the Fram Strait^15,16^. Sea ice from the Beaufort, Chukchi and East Siberian seas may become incorporated into the Beaufort gyre with some sea ice having the potential to exit this gyre and eventually join the Transpolar Drift^17^. Within the Arctic Ocean, sea ice dynamics are one of the key factors that potentially influences contaminant fate. Mobile sea ice floes in the Arctic Ocean are capable of entrapping contaminants along their drift pathways and playing a role in the redistribution of contaminants due to their eventual release upon melting of the ice^2^^,^^16,19^. Estimating backward sea ice trajectories is an important tool that facilitates an examination of sea ice sources, drift pathways, thickness changes and atmospheric processes acting on the ice cover^2,28–30^. When utilized in microplastic studies, backward sea ice trajectory data (i.e. sea ice sources and drift pathways) can be used to make inferences about potential regions where microplastic entrapment in sea ice occurred. The AWI ICETrack application, a lagrangian approach that traces sea ice backward in time using a combination of satellite-derived low-resolution drift products, is one available modelling approach that was utilised in the most recent study on microplastics in Arctic sea ice^2^. In the Arctic Ocean, sea ice has been identified as a temporal sink and secondary source of microplastics since contaminant release is projected to occur upon the melting of sea ice^1,2^. In the context of a changing climate, projections for Arctic sea ice include decreasing sea ice extent, reductions in sea ice thickness (less multi-year ice), alterations in the rate of sea ice drift, intensified melting of sea ice in the marginal zones and interruption of its Transpolar Drift^28,31–33^. These changing conditions will inevitably influence the dynamics of contaminant fate and transport in the Arctic Ocean, especially if the contaminants of interest are capable of being entrapped within, transported and subsequently released by sea ice.

Given that the 18 Arctic Large Marine Ecosystems (LMEs) support a diverse array of marine life^34^ and sea ice provides a range of microhabitats for numerous species^35–37^, an understanding of microplastic contamination in sea ice and surface waters of the Arctic Ocean is vitally important. The specific objectives of this study were to (i) provide a more spatially comprehensive assessment of microplastic concentration and composition in sea ice cores from the Arctic Ocean, (ii) assess the vertical distribution of microplastics in entire sea ice cores, (iii) estimate backward drift trajectories and identify source areas of sampled sea ice cores and (iv) assess microplastic abundance, distribution and composition in surface waters (beneath ice floes) in the Arctic Ocean.

## Results

### Quality control

Of the shipboard air contamination checks (n = 13) that were carried out, a single fibre (blue polyester fibre, 0.47 mm) was found. This indicates a low likelihood that microplastics were introduced to the samples as a result of airborne contamination. Of the method blanks (n = 15), 7 were free of contamination while the remainder contained either a single fibre (n = 6) or 2 fibres (n = 2). Fibres in the method blanks were polyester (n = 6), polyamide (n = 1) or a polyamide blend (n = 3). In order to account for any contamination that might have been introduced during ice core processing, a blank correction was applied whereby a single fibre was removed from each sub-section total. Additionally, if there were any matches between synthetic polymers found in the samples and those that came into contact with the samples (either during sampling or laboratory processing), these particles were excluded from the results.

### Microplastic contamination in sea ice cores

A total of 2031 particles were isolated from the 25 sea ice cores and analysed using FT-IR spectroscopy. Of these, 501 particles were rejected due to (i) poor spectral matches, (ii) matches with polymers used during sample collection or processing and, (iii) identification as being natural or semi-synthetic polymers. A further 117 synthetic polymers were excluded from further analyses since they were categorized as being macroplastics (>5 mm). Of the 1413 confirmed synthetic polymers, 223 were removed during the blank correction processes. Subsequent analyses are therefore based on 1190 synthetic polymers <5 mm from the sea ice cores.

**Figure 1:**
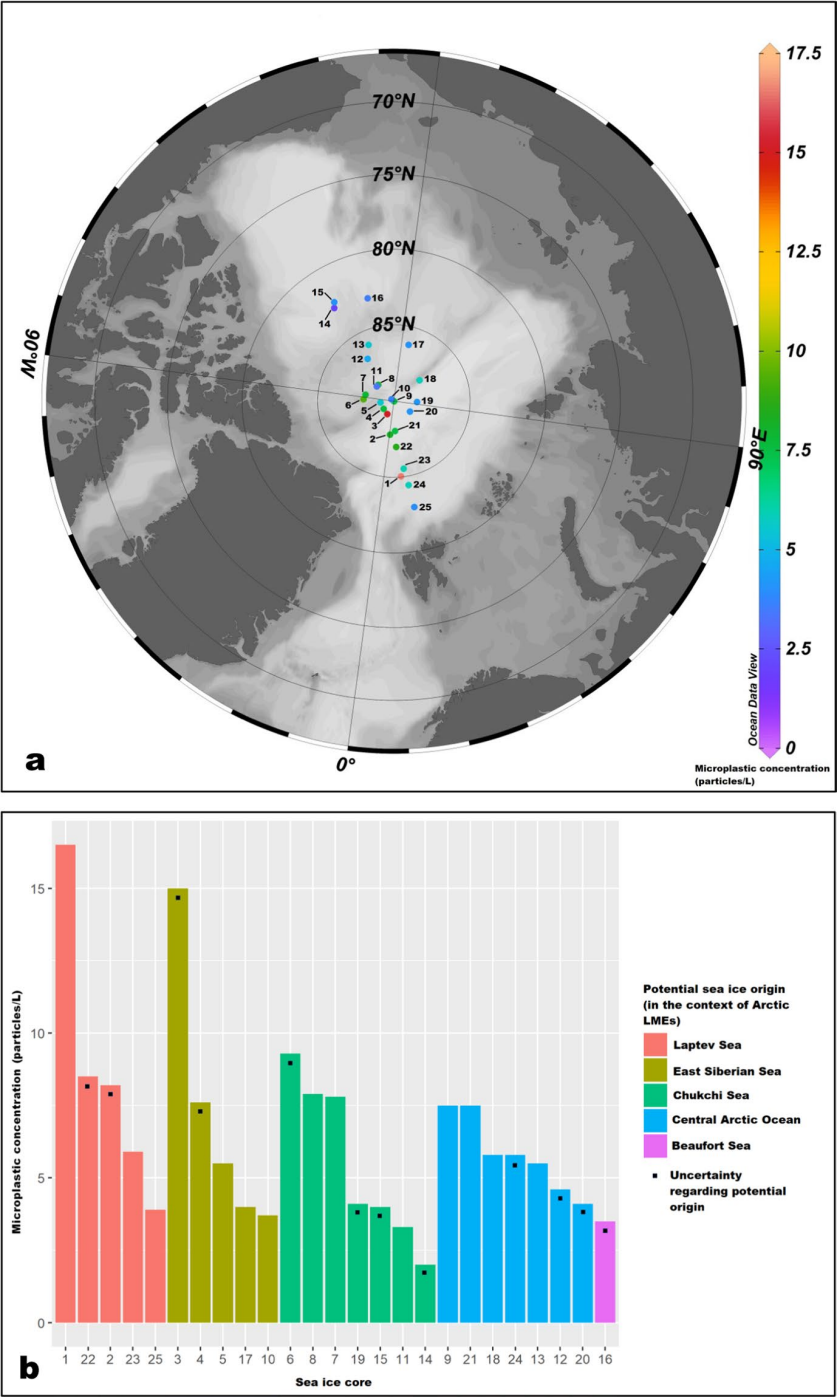
Microplastic concentration in sea ice cores from the Arctic Central Basin by sampling location (**a**) and potential origin (**b**). (**a**) generated using Ocean Data View^50^.

Microplastic concentration in sea ice cores (n = 25) from the Arctic Central Basin ranged between 2–17 particles L^−1^ with particle size ranging between 0.10 mm–4.99 mm (Fig. 1, Supplementary Table 1). Of the sampled cores, the majority had microplastic concentrations <8 particles L^−1^ (Fig. 1). The two cores with the highest microplastic concentrations were cores 1 (17 particles L^−1^) and 3 (15 particles L^−1^), (Fig. 1). In terms of polymer composition, a total of 9 types of synthetic polymers were found in the ice cores with an overall predominance of polyesters (57%) and polyamides (19%) followed by polyurethane (6%), styrene/acrylates (6%), polyacrylonitrile (6%), polyvinyl chloride (5%) and other polymers (1.3%) which included polypropylene and polyethylene (Supplementary Fig. 1). The majority of the microplastics that were found in the ice cores were fibres (79%) with the remainder (21%) being fragments. Size class distributions of microplastics in the ice cores were as follows: 0.1–0.5 mm (32%), 0.5–1 mm (20%), 1–2 mm (23%), 2–3 mm (13%), 3–4 mm (7%), 4–5 mm (4%), (Supplementary Fig. 1). In terms of colour, the majority of microplastics recorded in the sea ice cores were blue (53%) followed by red (10%), pink (9%), yellow (7%), black (5%), green (3.5%), transparent (3.5%), white (3%), grey (3%) and orange, purple and brown (3%).

### Vertical distribution of microplastics in sea ice cores

**Figure 2:**
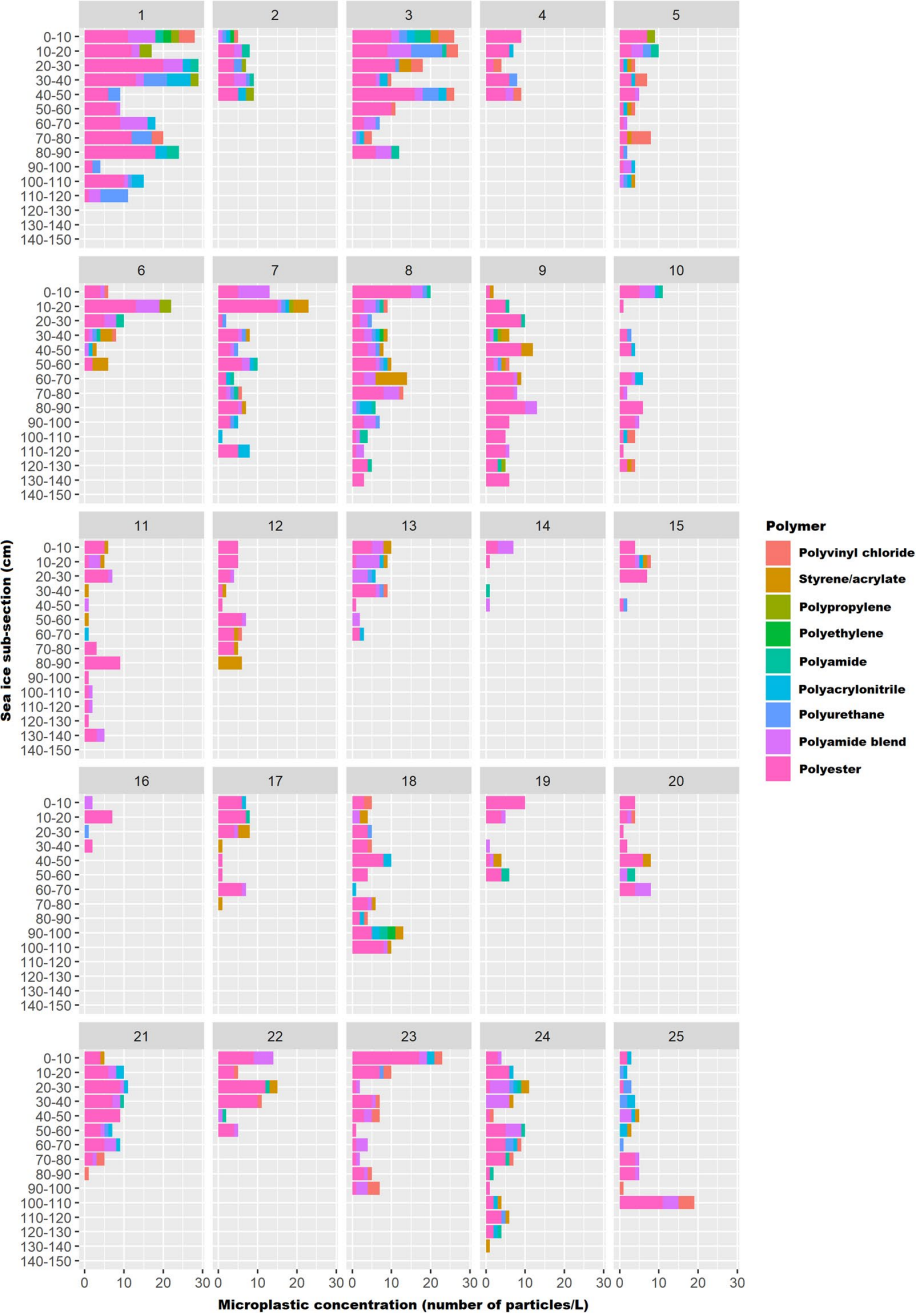
Vertical distribution of microplastics in sea ice cores from the Arctic Central Basin [In all cases (except cores 2, 8, 17, 20, 23), last bar indicative of sub-section of ice core in contact with the underlying seawater].

Overall, there appears to be no consistent pattern in the vertical distribution of microplastics within sea ice cores (Fig. 2), as well as no overall correlation between sub-section depth of the ice core and microplastic concentration (Spearman’s Rank Correlation, p-values > 0.05). However, when individual ice cores were considered, core 8 was the only ice core for which there was a significant negative correlation between microplastic concentration and sub-section depth (Spearman’s Rank Correlation, rho = −0.74, p-value = 0.001). For certain ice cores (3, 6, 7, 8, 23) it was also apparent that microplastic concentration was comparatively higher in certain upper sub-sections of the core (Fig. 2). Microplastics were also shown to be pervasive throughout the majority of the ice cores. In 4% (n = 9, total sub-sections = 232) of all ice core sub-sections, no microplastics were found (relevant to cores 8, 10, 14, 15, 17, 19, 23).

### Potential origin of sea ice

**Figure 3:**
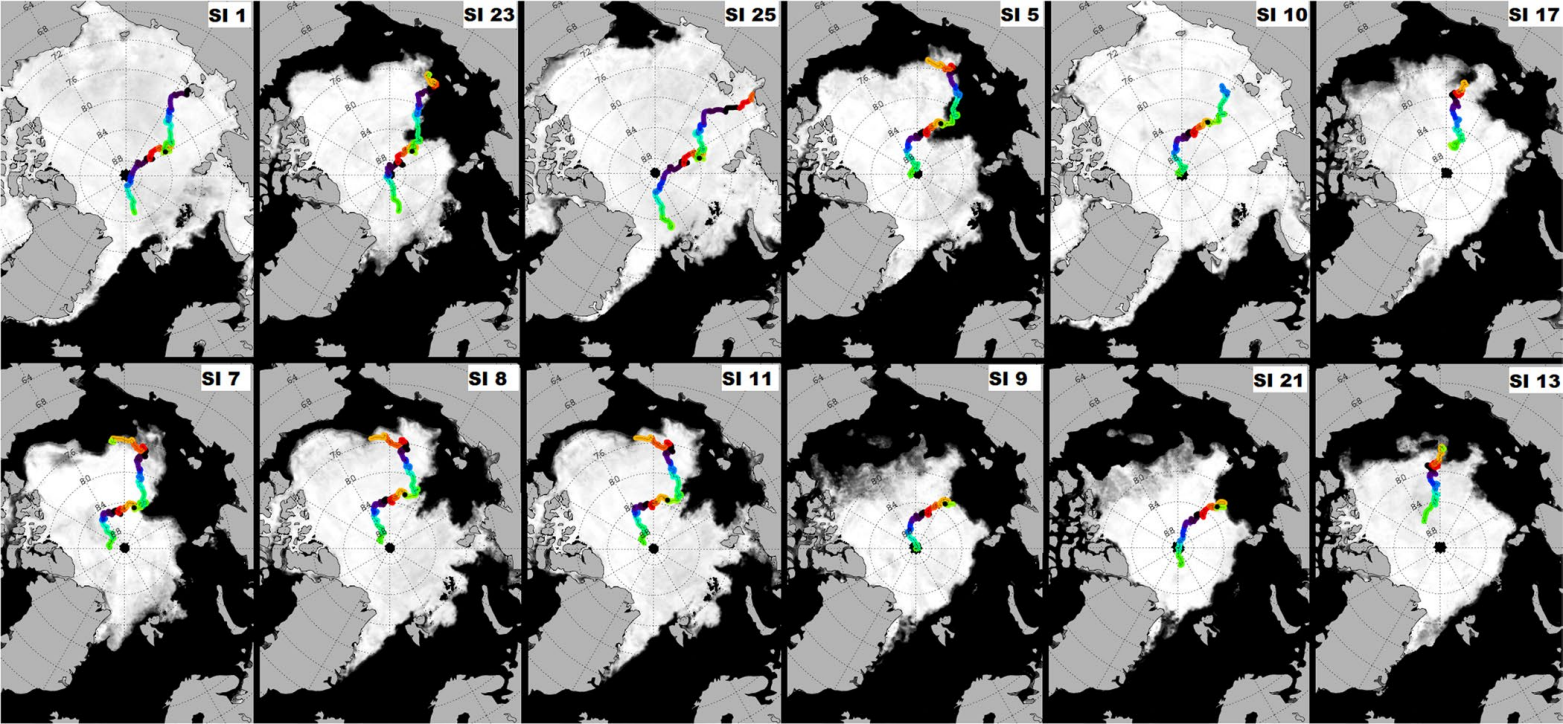
Backward trajectories derived using the AWI Ice Track application indicate formation zones for sampled sea ice in the (i) Laptev Sea (cores 1, 23, 25), (ii) East Siberian Sea (cores 5, 10, 17), (iii) Chukchi Sea (cores 7, 8, 11), (iv) Central Arctic Ocean (cores 9, 21, 13). [Trajectories shown here were for cores with a >75% match between model-predicted and field-recorded sea ice thickness, colours rep. months].

Backward trajectories of the ice cores indicated that they possibly originated from the (i) Siberian shelves in the Eurasian basin (i.e. Laptev Sea, East Siberian Sea), (ii) western Arctic (i.e. Beaufort Sea, Chukchi Sea) and, (iii) Central Arctic Ocean (Fig. 3, Supplementary Table 1). Of all the cores (n = 25), only 12 had a >75% match between measured sea ice thickness and model-predicted sea ice thickness (Fig. 3, Supplementary Table 1). Based on the backward trajectories of the sea ice cores, those cores which had the highest estimated microplastic concentrations i.e. >8 particles L^−1^ possibly originated in the Laptev, East Siberian and Chukchi Seas (Figs. 1, 3). Sea ice age estimations indicated that the majority of the sea ice cores were at least second year (SYI, n = 16, survived at least 2 melt seasons) and first year ice (FYI, n = 7, survived at least 1 melt season) with only two cores being classified as multi-year ice or greater than 3 years old (MYI, n = 2, survived at least 3 melt seasons), (Supplementary Table 1). Although univariate analyses indicated that there was a significant difference in microplastic concentrations between the different cores (Kruskal-Wallis, df = 24, p-value = 5.93 × 10^−8^), multivariate analyses (Principal Components Analysis), was unable to provide any useful discrimination between sea ice cores.

### Microplastics in seawater beneath ice floes

**Figure 4:**
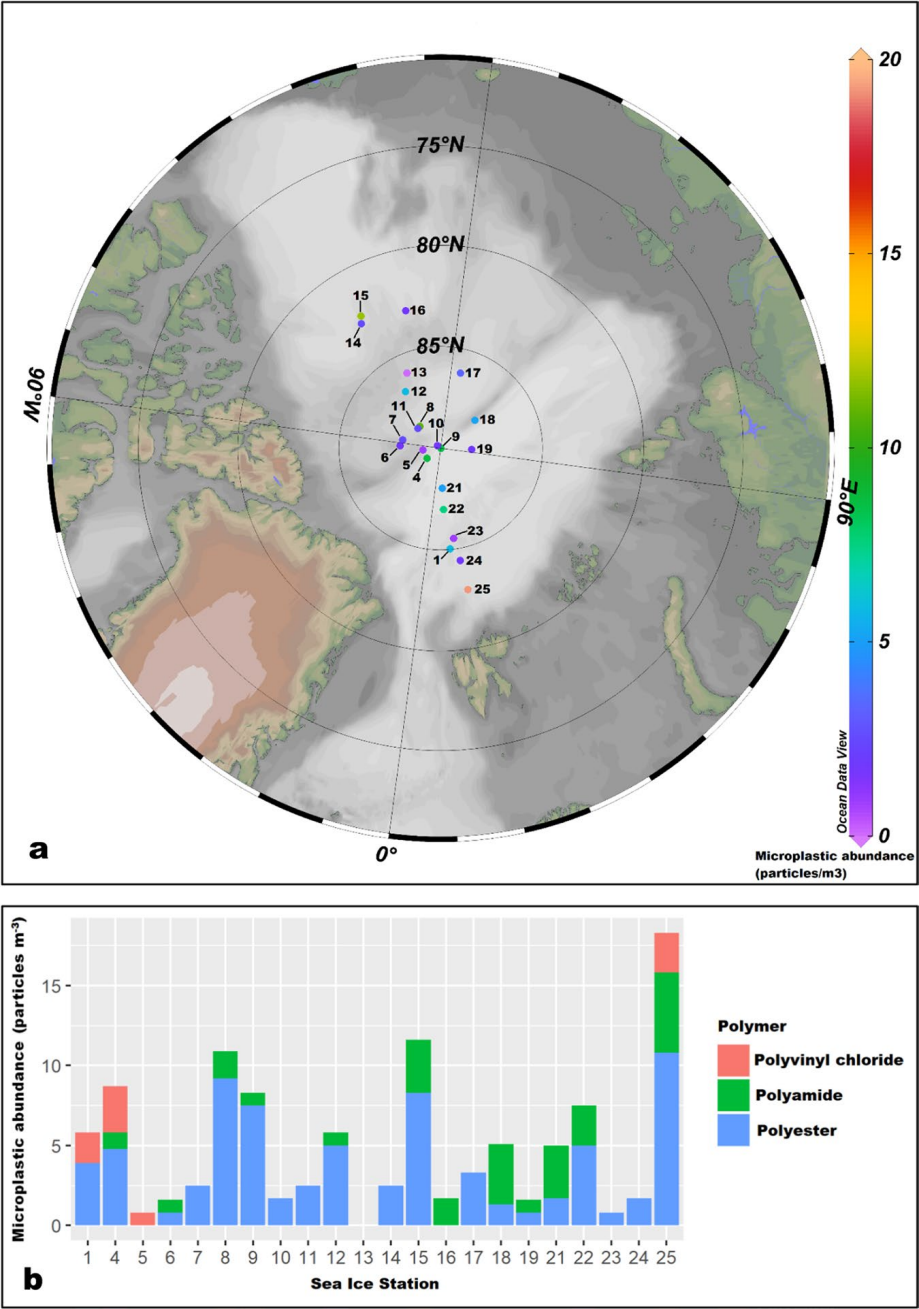
Microplastic abundance in surface waters beneath ice floes in the Arctic Central Basin. (**a**) generated using Ocean Data View^50^.

A total of 189 particles were isolated from surface water beneath ice floes samples in the ACB. Of these, 47 were rejected for the above-mentioned reasons (i.e. those stated for the ice core samples). A further 17 synthetic polymers were excluded since they were categorized as macroplastics (>5 mm). Subsequent analyses were based on 125 synthetic polymers <5 mm from the surface water samples. Microplastic abundance in seawater beneath the ice floes (0–18 particles m^−3^, Fig. 4) were orders of magnitude lower than those reported for sea ice, (2 × 10^3^) to (1.7 × 10^4^) particles m^−3^ (extrapolated from particles L^−1^). Of the 22 sites where surface waters were sampled, only one site did not have microplastics detected. In terms of polymer composition, the majority of polymers detected in surface waters of the ACB were polyesters (70%) and polyamides (23%) with a minority of polyvinyl chloride (7%). Microplastic size class distributions were as follows: 0.25–0.5 mm (16%), 0.5–1 mm (18%), 1–2 mm (34%), 2–3 mm (14%), 3–4 mm (11%), 4–5 mm (6%), with the majority of microplastics (68%) being <2 mm. Overall, fibrous microplastics predominated (89%). In terms of colour, the majority of microplastics were blue (58%) and red (18%), with other colours such as transparent (5%), pink (4%), yellow (4%), grey (4%), purple (3%), green (2%), black (2%) and brown (1%) also being recorded.

## Discussion

Microplastics, as evidenced by the present study, were ubiquitous in both sea ice cores and seawater underlying ice floes from the Arctic Central Basin (ACB). Microplastic concentrations in sea ice (estimations based on extrapolations from 25 sea ice cores, (2 × 10^3^) to (1.7 × 10^4^) particles m^−3^) were orders of magnitude higher than those reported for seawater beneath ice floes (0–18 particles m^−3^). While these findings corroborate those of previous studies^1,2^ that identified sea ice as a temporary sink for microplastics in the Arctic Ocean, it is collectively apparent that to date microplastic concentrations in Arctic sea ice have been underestimated. Analytical techniques (visual identification followed by FT-IR spectroscopy of potential microplastics) employed in the present study led to the exclusion of particles <100 µm from analysis and as such would have resulted in an underestimation of microplastic concentration in sea ice cores (number of cores = 25; range 2–17 particles L^−1^; extrapolated range (2 × 10^3^)–(1.7 × 10^4^) particles m^−3^). This is particularly relevant in light of the fact that the majority of microplastics in Arctic sea ice reported by a previous study^2^ using Imaging FTIR were <50 µm (number of cores = 5; extrapolated range (1.1 × 10^6^) – (1.2 ×10^7^) particles m^−3^). Although the previous study^2^ reported the highest microplastic concentrations thus far in sea ice, fibres were excluded from the analysis. By contrast, the present study reported a pre-dominance of fibrous microplastics (79%) in sea ice from the Arctic Ocean. It is therefore apparent that if either fibres or particles <100 µm are excluded from the findings, microplastic concentrations in sea ice will be underestimated. Furthermore, both the present study and a previous study^2^ showed that there was no consistent pattern in the vertical distribution of microplastics within sea ice cores. If isolated sub-sections of ice cores are used to estimate microplastic concentrations in Arctic sea ice as was done in a previous study (number of cores = 4, meltwater volumes 50–100 mL, extrapolated range 1.3–9.6 × 10^4^ particles m^−3^)^1^, those extrapolations will either be underestimations or overestimations of microplastic concentrations. In the present study, meltwater volumes in ice cores ranged between 3–12 L. Extrapolations from particles L^−1^ to particles m^−3^ are relevant for comparison to microplastic concentrations in surface and sub-surface waters. However, when microplastic concentrations from <20 L of meltwater from a single ice core is used to estimate microplastic concentrations in 1,000 L or 1 m^−3^ of meltwater, such estimations are based on numerous assumptions and limited datasets (few ice cores, low meltwater volumes). It is therefore suggested that microplastic concentrations in sea ice should be reported as particles L^−1^.

The identification of microplastic type (fibre, fragment, etc) and polymer composition is a critical component of microplastic studies as it provides investigators with some insight about the potential sources of microplastics in the environment. In the present study, 9 different types of synthetic polymers were reported in the sea ice cores while 3 were reported in surface waters underlying the ice floes. In both environmental compartments, the majority of microplastics were comprised of fibrous polyesters (57%- sea ice cores, 70%- surface waters) and polyamides (19%- sea ice cores, 23%- surface waters). Both polyesters and polyamides have a higher density than seawater^38^, thus raising the question as to why these particles were prevalent in both the sea ice cores and surface waters of the Arctic Central Basin. These findings were comparable to the first published study on microplastics in Arctic sea ice^1^ whereby it was reported that the most common synthetic polymers were polyesters (21%) and polyamides (16%). By contrast, although another study^2^ on microplastics in Arctic sea ice did report on the presence of polyester and polyamide, the most dominant synthetic polymer in the examined cores was polyethylene (48%) with the difference in findings possibly occurring due to the exclusion of fibres. In the western Arctic at the Bering and Chukchi Seas^8^, polyesters predominated in surface waters. In the present study, the majority of microplastics in sea ice cores (79%) and surface waters (89%) were fibrous. Similarly, in the Bering and Chukchi Seas^8^, fibrous microplastics were predominant in surface waters. In the marine environment, fibres may be originating from fishing gear, textiles due to laundering fabric and cigarette filters^38^. Laboratory experiments have indicated that the input of textile fibres into the marine environment can occur following the discharge of wastewater from domestic washings^38–43^. Recently, it was reported^14^ that both microplastics and microfibers were present in snow from ice floes in the Fram Strait which suggests that these particles could have been transported into the region by winds and been deposited onto ice floes and surface waters via snow. For the remote Arctic Ocean, it is difficult to pinpoint the exact origin of fibrous microplastics. Although definitive statements cannot be made about the origin of microplastics in surface waters or sea ice of the ACB, potential sources of these contaminants may include (i) riverine discharge from the Siberian and Canadian rivers^7^, (ii) influx of contaminated Pacific and Atlantic waters^1^, (iii) grey water discharge from vessels operating in the Arctic^7^ and, (iv) atmospheric deposition^14^.

Similar to the situation for sediments and other contaminants^15–27^, microplastic entrapment in Arctic sea ice potentially occurs during its initial formation over both the shallow marginal shelves of the Arctic Ocean and the deep central basin. In the present study, backward drift trajectories estimated that the sea ice floes from which the ice cores were sampled originated from the Siberian shelves in the Eurasian Basin, western Arctic and central Arctic. If microplastics were entrapped during sea ice formation in these areas and ice floes survived subsequent melt seasons, microplastic composition in sampled sea ice cores could be reflective of the microplastic composition in surface waters from these areas. At the marginal shelves of the Arctic, freshwater discharge from Siberian rivers; Dvina and Pechora (Barents Sea), Ob and Yenisei (Kara Sea), Lena (Laptev Sea) and Kolyma rivers (East Siberian Sea), and the Canadian Mackenzie river (Beaufort Sea)^34,44^, could be potential contributors to the microplastic load in Arctic sea ice. Since anthropogenic activities occur within the catchment areas of these rivers, the influx of freshwater into the Arctic basin is a potential pathway via which contaminants could enter this marine ecosystem^45,46^. Presently however, the contribution of riverine discharge to the microplastic load in Arctic sea ice remains uncertain due to the absence of field data. Pacific water influx into the Arctic Ocean through the Bering Strait also represents a potential source of contamination^2^ in that Pacific waters are influenced by anthropogenic activities that occur in northern America and eastern Russia. The Chukchi, Beaufort and East Siberian Seas which border the central Arctic are influenced by Pacific waters^8^ and thus any sea ice formed in these seas could potentially reflect microplastics that are present in the surface waters in these areas. The backtracking results that were presented in the present study must be interpreted with caution since there was a significant mismatch (>75%) between field and model-predicted ice thickness for 10 of the 25 ice cores (Supplementary Table 1). Although the AWI IceTrack application which generated these backward trajectories was validated using reconstructed pathways of real buoys, matches between ice thickness in the field and model-predicted ice thickness generally provide an indication of the reliability of the findings. The ice thickness mismatches could have been influenced by the fact that the tracking algorithm reconstructs the movement and evolution of sea ice that is mainly found in the Arctic Ocean and does not resolve dynamics and formation of new ice in leads (which is possibly where these specific ice cores were retrieved).

Microplastic incorporation into sea ice floes can also potentially occur during its drift across the central Arctic. Backward drift trajectories produced for sea ice sampled in the present study indicated that 24 of the 25 sea ice cores survived at least one summer melt and endured at least one winter period and that there was either drift across or initial sea ice formation within the ACB. These findings suggest that microplastics found in the sampled sea ice cores were potentially reflective of microplastics in surface waters of the ACB. Once present in surface waters of the central Arctic, periods of freezing will facilitate microplastic entrapment in newly formed ice or on the underside of existing ice floes. Microplastic presence in waters beneath ice floes (0–18 particles m^−3^) and sub-surface waters of the central Arctic^7^ indicates the availability of these particles for incorporation into sea ice, whether that is first year ice forming over the central basin or vertical ice growth on the underside of the floes. In the western Arctic^8^, microplastic abundance in surface waters of the Bering Sea ranged between 0.035–0.26 particles m^−3^ and in the Chukchi Sea between 0.086–0.31 particles m^−3^. Outside the central Arctic, microplastic abundance in surface Arctic waters south and southwest of Svalbard^3^ ranged between 0–1.3 particles m^−3^ and east of Greenland^4^ it ranged between 0.2–2.6 particles m^−3^ in 2005 and 0.8–4.5 particles m^−3^ in 2014. While conclusive statements cannot be made about differences in the reported microplastic abundances, it is plausible that sea ice functions as a secondary source of microplastics in the central Arctic and contributes to higher microplastic abundances. During transport, atmospheric deposition of microplastics unto the surface of an ice floe may also occur. A recent study^14^ reported that microplastics and microfibers were present in snow retrieved from ice floes in the Fram Strait. These findings suggested that these particles were potentially transported into the Arctic by winds and deposited unto the ice floes via snow^14^. Atmospheric fallout of microplastics suggests that these contaminants can be transported through the atmosphere and reach remote areas^14,47,48^. It is possible that microplastics can be transported into the Arctic region by winds and that these particles can be deposited either unto ice floes during transport or directly unto surface waters^14^.

Arctic sea ice is not the ultimate but an intermediate sink for microplastics. From a temporal perspective, seasonal environmental conditions (spring/summer melt seasons) influence sea ice melting and subsequent microplastic release in the Arctic Ocean. During summer, snow and ice melt off the upper portion of the ice floe with meltwater either running off the ice floe, percolating into the surface of the floe, accumulating in melt ponds or refreezing on the underside^16^. Summer melt periods can therefore facilitate the redistribution of microplastics within an ice floe in that particles that were present in the lower layers of an ice floe may eventually make their way to the top layer of an ice floe. In the present study, there was no consistent pattern in the vertical distribution of microplastic within the sea ice cores. Some of the sea ice cores, however, reflected comparatively higher concentrations of microplastics in the upper sub-sections and it is postulated that re-distribution processes may have resulted in this pattern. Of note is the fact that sea ice melting in the Arctic Ocean usually coincides with bursts of biological activity, i.e. ice algal blooms in spring and phytoplankton blooms in summer^35^. Sea ice in the Arctic Ocean functions as a key habitat for numerous species of marine organisms^32–37^. Presently, it remains uncertain whether microplastics are incorporated within the ice crystal structure or brine channels of sea ice and whether microplastics may pose a threat to sea ice meiofauna or in-ice fauna that inhabit sea ice. Marine organisms that live in close association with sea ice, specifically under-ice or sea ice macrofauna and sub-ice fauna, are likely to interact with microplastics once these particles are released into the underlying seawater from melting ice. In the Arctic Ocean, dominant under-ice fauna are the gammarid amphipods while sub-ice fauna include various species of copepods and fish such as the polar cod (*Boreogadus saida*) and Arctic cod (*Arctogadus glacialis*)^37^. Recently, microplastic fragments were reported in the stomachs of polar cod sampled from waters in the Eurasian Basin of the Arctic Ocean and north of Svalbard^13^ and in the digestive tracts of polar cod sampled in Arctic waters east of Greenland^6^. Based on the fact that interactions can potentially occur between marine organisms and microplastics in the Arctic Ocean, laboratory experiments are needed to elucidate the impact of those interactions with polar organisms. From a spatial perspective, regions which could be most at risk from an influx of contaminants (e.g. microplastics) from melting sea ice include nearshore areas over the marginal shelves, the central Arctic basin and the marginal ice zone e.g. Fram Strait, Barents Sea^16,19,21,31^.

The Arctic Ocean is a dynamic ecosystem where projections for Arctic sea ice in the context of a changing climate can inevitably influence contaminant fate and transport, especially if the contaminants of interest are capable of being entrapped within, transported and subsequently released by sea ice. Corroborating previous studies^1,2^, the present study evidenced that sea ice is capable of functioning as an intermediate sink, secondary source and a transport medium for microplastics in the Arctic Ocean. For the first time, microplastic presence in seawater underlying ice floes was reported for the central Arctic. Understanding the presence, sources, transport pathways and fate of microplastics in the Arctic Ocean is critical to determining the potential threats posed by such contaminants to marine organisms that inhabit or depend upon different environmental compartments in this ecosystem.

## Methods

### Sample collection

This study was conducted onboard Swedish icebreaker Oden during the Arctic Ocean 2016 expedition (August 8^th^ to September 19^th^ 2016). At 25 ice stations, sea ice cores (n = 25) were retrieved and seawater (n = 22) was pumped from beneath the ice and filtered onsite for microplastics. At each ice station, a suitable location upwind of all other site activities was selected and overlying snow was removed from an area of approximately 0.5 m^2^. Ice cores were collected using a Nordic ice drill with an attached Husqvarna X-series 326A125 motor and a stainless-steel core barrel of 12.5 cm diameter. At all sites, the goal was to completely penetrate the ice by drilling and reach the underlying seawater. A single ice core (n = 1) was retrieved at each site, placed into clean bags (polyethylene) and transported to the laboratory onboard the vessel for further processing. Once the sea ice core was retrieved at a site, water was then pumped from under the ice floe. A pre-cleaned polyvinyl chloride (PVC) hose (approximately 2 m in length) was inserted into the drilled hole. This hose was then connected to the inlet of a manual JABSCO Amazon Warrior pump (Model Number 29280-0000). Another hose of approximately 1 m in length was then connected to the outlet of the pump. Prior to any further connections, seawater was pumped from beneath the ice to flush the system of any contaminants. At this point, the flow rate of water through the system was manually checked by pumping seawater into a 1 L measuring cylinder. This was performed in triplicate in order to estimate the length of time needed for pumping the relevant volume of water. Following this, the hose from the outlet of the pump was positioned into the cover of a wooden stand containing a stainless-steel sieve (250 μm). This wooden stand with the sieve was positioned over a bucket into which filtered water flowed. The water that entered the bucket exited the site via a hose that was approximately 3 m in length. At 22 (of the 25) ice stations, water was manually pumped for at least 40 minutes thus ensuring that 1200 L of water was pumped at all sites, with the exception of two sites at which lower volumes (780 L, 1036 L) were pumped. Once pumping of water from beneath the ice was completed, the sieve was covered with aluminium foil, secured in the wooden stand and transported to the laboratory for further processing. At 3 (of the 25) ice stations, seawater could not be pumped from beneath the ice floe due to the incomplete penetration of the ice floe during drilling.

### Laboratory processing and analyses

The outer surface of each ice core was scraped off using a boomerang scraper. A stainless-steel hand saw was then used to cut each ice core into 10 cm vertical subsections. Subsections were placed into individual clean Ziploc bags (polyethylene) and allowed to melt for 24–48 hours. Once melted, the water from each subsection was transferred to a graduated cylinder and its volume measured. Each Ziploc bag was rinsed in triplicate with Milli-Q water to ensure that all particles were transferred out of the bag. Water from each sub-section was filtered under vacuum onto glass microfiber paper (GF/C); Whatman: 47 mm, pore size: 1.2 μm, using a Buchner funnel and a vacuum flask. Each filter paper was folded, placed into an aluminium foil packet and stored in a freezer (−20 °C) until further processing. The sieve that was used for filtering water from beneath the ice was rinsed in triplicate and all water was also processed in the same manner (vacuum filtration of water and collection of particles onto glass microfiber paper). Potential microplastics were identified and isolated based on a previously described method^49^. Briefly, individual filter papers were visually examined under a dissecting microscope (Olympus SZX10) equipped with a polariser and camera (Q Imaging Retiga 2000R) and potential microplastics were identified based on characteristic features^49^. Potential microplastics from each sample were photographed, described according to form (fibre, fragment, etc) and length measurements were taken prior to transferring to a clean filter paper. Potential microplastics were assigned to two broad categories (fibres, fragments) and to six length categories: 0.1–0.5 mm, 0.5–1.0 mm, 1.0–2.0 mm, 2.0–3.0 mm, 3.0–4.0 mm, 4.0–5.0 mm. Filter papers with potential microplastics from each sample were stored in clean, labelled petri dishes. All potential microplastics were analysed by Fourier transform infrared (FT-IR) spectroscopy on a Bruker Vertex 70 Infrared Spectrometer coupled to a Hyperion 1000 microscope according to a previously described method^49^. Briefly, each sample spectrum was compared with those of known standard polymers in the (i) Bruker Optics Attenuated Total Reflectance (ATR) Polymer and (ii) Synthetic Fibres ATR libraries. Samples which produced spectra with a match <60% were automatically rejected. All remaining spectra (>60%) were individually examined to ensure that there was clear evidence of peaks from the sample corresponding to known peaks of standard polymers. Overall, matches with >70% similarity were accepted while some between 60–70% similarity were accepted.

### Method validation and contamination prevention

Several measures were taken to minimise contamination of samples. In the field, (i) microplastic sampling was conducted upwind of all other activities, (ii) nitrile gloves were used when handling ice cores, (iii) the manual pump used at the ice stations was flushed with water prior to pumping seawater, and (iv) the stainless steel sieve that was used at the ice stations had a wooden cover affixed to it during filtration. In the lab, (i) ice processing was conducted on a wooden surface, (ii) the wooden work area was washed down with Milli-Q water in between processing of individual ice core subsections, (iii) all equipment (scraper, saw) was washed with MilliQ water, (iv) lab coats, cotton clothing and gloves were worn during sample processing, and (v) all containers used during sample processing were cleaned using Milli-Q water. Checks were conducted to determine whether there was any contamination during sample processing. Clean petri dishes with filter paper were left exposed to the air during ice core and laboratory processing to determine if there was any airborne contamination. To determine whether there was any additional contamination during the processing of the melted sea ice, method blanks were conducted. For each method blank, 750 mL of Milli-Q water was placed into clean Ziploc bags and left for 24–48 hours. This water then underwent the exact processing as would have occurred for water from an ice core sub-section.

### Data analyses

Source areas and backward drift trajectories for the sea ice cores were estimated using the AWI ICETrack application. The lagrangian approach traces sea ice backward in time using a combination of satellite-derived low-resolution drift products^28^. So far, ICETrack has been used in a number of publications to examine sea ice sources, pathways, thickness changes and atmospheric processes acting on the ice cover cover^2,29,30^. For each ice core, input to the application included sampling date and location (latitude, longitude) while output included a plot of the estimated pathway as well as ancillary data associated with the pathway. Predicted sea ice thickness by a thermodynamic model and measured sea ice thickness was compared in order to assess the validity of the findings for the various sea ice cores. All maps were generated using Ocean Data View (ODV) Version 4.7.10^50^ and all graphs were generated using R version 3.4.4^51^. Univariate analyses were conducted to determine whether (i) there were significant differences in microplastic concentrations among the sea ice cores (Kruskall-Wallis) and, (ii) there were significant correlations between microplastic concentration and sub-section depth of the ice cores (Spearman’s Rank Correlation). Multivariate analysis, i.e. Principal Components Analysis (PCA), was conducted to provide further discrimination between the ice cores.

## Supplementary information

Supplementary Information.

## Data Availability

All data related to this study is available without restriction.
