# Supplementary material for: Microplastics in sea ice and seawater beneath ice floes from the Arctic Ocean
Source: Sci Rep. 2020 Mar 19;10:5004. doi: 10.1038/s41598-020-61948-6 (PMC7081216; doi:10.1038/s41598-020-61948-6)
Supplement: Supplementary file 1 — Supplementary Information. [file 41598_2020_61948_MOESM1_ESM.docx]

**Supplementary Information**

**Manuscript Title: Microplastics in sea ice and seawater beneath ice floes from the Arctic Ocean**

La Daana K. Kanhai^a, b*^, Katarina Gardfeldt^c^, Thomas Krumpen^d^, Richard C. Thompson^b^, Ian O’Connor^a^

^a^Marine and Freshwater Research Centre, Galway Mayo Institute of Technology, Galway, Ireland

^b^Marine Biology and Ecology Research Centre, University of Plymouth, Plymouth, United Kingdom

^c^Department of Chemistry and Chemical Engineering, Chalmers University of Technology, Göteborg, Sweden

^d^Alfred Wegener Institute, Helmholtz Centre for Polar and Marine Research, Bremerhaven, Germany

Supplementary Table 1: Model-predicted data (age, ice type, ice thickness, formation zone) for the sea ice cores based on retrieval date and location

| **Core** | **Latitude** | **Longitude** | **Sampling date** | **No. of microplastics** | **Meltwater volume (L)** | **Microplastic**  **concentration (number of particles/L)** | **Sea ice age**  **(days)** | **Type of ice** | **Core length**  **(cm)** | **Model predicted thickness (cm)** | **Difference between core length & model predicted thickness (%)** | **Potential Origin** |
| --- | --- | --- | --- | --- | --- | --- | --- | --- | --- | --- | --- | --- |
| 1 | 85.0414 | 12.9208 | 13/08/2016 | 117 | 7.1 | 16.5 | 581 | SYI | 118 | 113 | 4 | LS |
| 2 | 87.7889 | 0.9455 | 14/08/2016 | 32 | 3.9 | 8.2 | 641 | SYI | 48 | 116 | NA | LS |
| 3 | 87.7889 | 0.9455 | 15/08/2016 | 108 | 7.2 | 15.0 | 692 | SYI | 85 | 124 | 46 | ESS |
| 4 | 89.0468 | -19.3978 | 15/08/2016 | 30 | 3.9 | 7.6 | 694 | SYI | 48 | 125 | 160 | ESS |
| 5 | 89.1639 | -45.5045 | 17/08/2016 | 47 | 8.6 | 5.5 | 684 | SYI | 113 | 116 | 3 | ESS |
| 6 | 89.1245 | -76.5614 | 18/08/2016 | 38 | 4.1 | 9.3 | 703 | SYI | 56 | 123 | 120 | CS |
| 7 | 88.0025 | -85.9708 | 19/08/2016 | 76 | 9.7 | 7.8 | 697 | SYI | 120 | 118 | 2 | CS |
| 8 | 88.0952 | -94.9308 | 20/08/2016 | 98 | 12.3 | 7.9 | 674 | SYI | 147 | 114 | 22 | CS |
| 9 | 88.5268 | -128.7235 | 21/08/2016 | 89 | 11.9 | 7.5 | 348 | FY1 | 135 | 105 | 22 | CAO |
| 10 | 89.9885 | 48.4153 | 22/08/2016 | 41 | 11.1 | 3.7 | 479 | SY1 | 125 | 103 | 18 | ESS |
| 11 | 89.7980 | -120.0168 | 24/08/2016 | 37 | 11.1 | 3.3 | 678 | SYI | 137 | 114 | 17 | CS |
| 12 | 88.5182 | -123.2861 | 26/08/2016 | 31 | 6.7 | 4.6 | 1422 | MYI | 83 | 144 | 73 | CAO |
| 13 | 86.7316 | -140.4501 | 29/08/2016 | 31 | 5.6 | 5.5 | 341 | FYI | 75 | 84 | 12 | CAO |
| 14 | 85.9559 | -148.1900 | 30/08/2016 | 8 | 4.0 | 2.0 | 343 | FYI | 53 | 96 | 81 | CS |

NA-Not available, core length recorded not representative of ice thickness as ice was not penetrated by drilling; FYI-First Year Ice; SYI-Second Year Ice; MYI-Multi-year Ice;

BS-Beaufort Sea; CAO-Central Arctic Ocean; CS-Chukchi Sea; ESS-East Siberian Sea; LS-Laptev Sea.

Supplementary Table 1: Model-predicted data (age, ice type, ice thickness, formation zone) for the sea ice cores based on retrieval date and location

| **Core** | **Latitude** | **Longitude** | **Sampling date** | **No. of microplastics** | **Meltwater volume (L)** | **Microplastic**  **concentration (number of particles/L)** | **Sea ice age**  **(days)** | **Type of ice** | **Core length**  **(cm)** | **Model predicted thickness (cm)** | **Difference between core length & model predicted thickness (%)** | **Potential Origin** |
| --- | --- | --- | --- | --- | --- | --- | --- | --- | --- | --- | --- | --- |
| 15 | 82.7416 | -139.8267 | 02/09/2016 | 18 | 4.5 | 4.0 | 355 | FYI | 51 | 92 | 80 | CS |
| 16 | 82.4130 | -141.3739 | 03/09/2016 | 11 | 3.1 | 3.5 | 334 | FYI | 38 | 89 | 134 | BS |
| 17 | 83.0300 | -158.1485 | 05/09/2016 | 31 | 7.7 | 4.0 | 336 | FYI | 87 | 83 | 5 | ESS |
| 18 | 86.1915 | 172.7474 | 07/09/2016 | 51 | 8.8 | 5.8 | 1440 | MYI | 109 | 141 | 29 | CAO |
| 19 | 87.8334 | 136.6409 | 08/09/2016 | 20 | 4.9 | 4.1 | 697 | SYI | 59 | 104 | 76 | CS |
| 20 | 88.4850 | 95.3654 | 10/09/2016 | 20 | 4.8 | 4.1 | 368 | SYI | 64 | 97 | NA | CAO |
| 21 | 88.7405 | 64.7840 | 11/09/2016 | 62 | 8.3 | 7.5 | 376 | SYI | 104 | 102 | 2 | CAO |
| 22 | 88.0378 | 9.9060 | 12/09/2016 | 44 | 5.2 | 8.5 | 578 | SYI | 59 | 102 | 73 | LS |
| 23 | 86.9902 | 10.3032 | 13/09/2016 | 49 | 8.3 | 5.9 | 717 | SYI | 111 | 116 | 5 | LS |
| 24 | 85.5214 | 15.6777 | 14/09/2016 | 67 | 11.6 | 5.8 | 14 | FYI | 150 | 12 | 92 | CAO |
| 25 | 84.4055 | 17.4587 | 15/09/2016 | 34 | 8.8 | 3.9 | 669 | SYI | 113 | 112 | 1 | LS |

NA-Not available, core length recorded not representative of ice thickness as ice was not penetrated by drilling; FYI-First Year Ice; SYI-Second Year Ice; MYI-Multi-year Ice; BS-Beaufort Sea; CAO-Central Arctic Ocean; CS-Chukchi Sea; ESS-East Siberian Sea; LS-Laptev Sea.

a

Supplementary Figure 1: Polymer composition (a) and size class distribution (b) in sea ice cores from the Arctic Central Basin.
